# Rectified Latent Variable Model-Based EMG Factorization of Inhibitory Muscle Synergy Components Related to Aging, Expertise and Force–Tempo Variations

**DOI:** 10.3390/s24092820

**Published:** 2024-04-28

**Authors:** Subing Huang, Xiaoyu Guo, Jodie J. Xie, Kelvin Y. S. Lau, Richard Liu, Arthur D. P. Mak, Vincent C. K. Cheung, Rosa H. M. Chan

**Affiliations:** 1 Department of Electrical Engineering, City University of Hong Kong, Hong Kong, China; subing.huang@my.cityu.edu.hk (S.H.); xiaoyu.guo@my.cityu.edu.hk (X.G.); richliu@hku.hk (R.L.); 2 School of Biomedical Sciences, The Chinese University of Hong Kong, Hong Kong, China; jodiexie@cuhk.edu.hk (J.J.X.); yatsingkelvinlau@cuhk.edu.hk (K.Y.S.L.); vckc@cuhk.edu.hk (V.C.K.C.); 3 Gerald Choa Neuroscience Institute, The Chinese University of Hong Kong, Hong Kong, China; 4 Department of Psychiatry, Faculty of Medicine, The Chinese University of Hong Kong, Hong Kong, China; arthurdpmak@cuhk.edu.hk; 5 Cambridgeshire and Peterborough NHS Foundation Trust, Fulbourn Hospital, Cambridge CB21 5EF, UK

**Keywords:** muscle synergy, rectified latent variable model, inhibitory, motor control, piano playing

## Abstract

Muscle synergy has been widely acknowledged as a possible strategy of neuromotor control, but current research has ignored the potential inhibitory components in muscle synergies. Our study aims to identify and characterize the inhibitory components within motor modules derived from electromyography (EMG), investigate the impact of aging and motor expertise on these components, and better understand the nervous system’s adaptions to varying task demands. We utilized a rectified latent variable model (RLVM) to factorize motor modules with inhibitory components from EMG signals recorded from ten expert pianists when they played scales and pieces at different tempo–force combinations. We found that older participants showed a higher proportion of inhibitory components compared with the younger group. Senior experts had a higher proportion of inhibitory components on the left hand, and most inhibitory components became less negative with increased tempo or decreased force. Our results demonstrated that the inhibitory components in muscle synergies could be shaped by aging and expertise, and also took part in motor control for adapting to different conditions in complex tasks.

## 1. Introduction

How the central nervous system (CNS) controls the muscles to complete variant motor tasks has been a long-term research topic in the field. Considering the high number of degrees of freedom, it would cause an extremely heavy burden on the CNS if the muscles were individually controlled. One common opinion proposes that the CNS controls the muscles through a linear combination of neuromotor building blocks named muscle synergies to reduce the computational complexity [1,2,3,4,5,6,7]. Muscle synergies are defined as sets of muscles coordinately activated that work as a fixed combination [8,9,10,11,12]. The theory has been widely applied in fields including sports training, clinic assessment and diagnosis [13,14,15,16,17,18,19,20,21].

The neural bases of muscle synergies have been demonstrated by multiple animal experiments. Results of micro-stimulation and transection experiments on the spinal cord of frogs and rodents supported the existence of muscle synergies encoded in the spinal cord [22,23,24]. T. Takei and K. Seki’s research on monkeys during a precision grip task further revealed that spinal interneurons contribute to the coordination of multiple finger muscles in the control of hand grasping in primates [25,26]. Literally, each premotor neuron in the spinal cord innervates multiple muscles, known as its muscle field [27], and the spinal premotor interneurons (PreM-INs) were found to be distributed as clusters instead of being uniformly distributed across hand muscles. The clusters were further revealed to correspond to the muscle synergies factorized from electromyography (EMG) signals, demonstrating that PreM-INs play an important role in muscle synergy generation [28]. PreM-INs were found to have significant postspike effects [25,29], which could be either facilitations or suppressions [25,30]. Suppressions may introduce inhibitory components into muscle synergies, which remain undetectable and unresolvable using the widely employed non-negative matrix factorization (NMF) technique. This limitation prompts further investigation into how various factors, including aging, might influence suppression with new computational methods [31].

Aging, as a life stage everyone would experience, has been revealed to influence spinal motor neurons [32] and alter muscle synergies [33]. Thus, it could be inferred that there could be influence from aging on the inhibitory components as well. Understanding the influence could significantly enhance our understanding about aging in the motor system. Meanwhile, training is a necessary process to master new skills, and prior research has proposed the shaping of structures and balances between muscle components within synergies during motor skill learning [18,34]. One of the purposes of training and the hallmark of expertise is the ability to adapt to variant constraints when completing the tasks [35,36]. However, current related research studies focused on the positive components and left the modulation on inhibitory components to be explored. Research with *Xenopus* tadpoles [37] proposed changes in the firing reliability in some inhibitory interneurons when their locomotor speed slows down. Thus, it is highly plausible that the inhibitory components in muscle synergies also function and are fine-tuned to help adapt to the variant constraints and requirements. However, the precise mechanisms by which these inhibitory components are modulated and how these components contribute to motor control remain to be elucidated.

To address the two questions mentioned above—decoding the inhibitory components in muscle synergies and revealing their modulation and functionality in aging, motor training, and adaptation to different task constraints—we have designed a specific experimental protocol and introduced a synergy extraction algorithm distinct from the commonly used NMF. We employed piano playing, a complex motor task involving variant tempo–force requirements, as the experiment task, and invited expert pianists from a range of ages and skill levels. To detect the inhibitory components, we utilized a rectified latent variable model (RLVM) that does not impose non-negative constraint on muscle synergies for synergy extraction [38]. The model has achieved robust excellent performance for neuron data [39,40,41]. The research is insightful in that it would help improve our understanding of the mechanism of human motor control, as well as propose a possible direction to study the inhibitory effects of motor neurons in an easier way. Furthermore, the modulation of inhibitory components could shed light on robotic control by introducing inhibitory signals to achieve more precise and stable control.

## 2. Materials and Methods

### 2.1. Participant Information

Completing the performance fluently under the requirements with which the players were not at all familiar could be challenging, especially when it came to the pieces. We thus invited ten experienced pianists to be the participants, labeled from Exp01 to Exp10. They all had expert-level certificates or music degrees, or were professional pianists with concert experience. We selected participants with certified skills and over 10 years of training experience.

For aging-related analysis, ten subjects were divided into three groups: a young group with subjects under 30 years of age (four subjects), a middle-aged group with subjects between 30 and 50 years of age (five subjects), and an older group with subjects over 50 years of age (one subject). For expertise-related analysis, ten subjects were divided into two groups: a junior expert group (three subjects) and a senior expert group (five subjects). The “junior experts” had performer’s certificates on piano but at a lower level, such as The Associate of the Royal Schools of Music (ARSM) and Licentiate of the Royal Schools of Music (LRSM) diplomas of the Associated Board of the Royal Schools of Music (ABRSM), Licentiate of Trinity College of London (LTCL) of Trinity College London, or had a bachelor’s degree in music. The “senior experts” had performer’s certificates at the highest level in music exams, such as The Fellowship of the Royal Schools of Music (FRSM) diploma of ABRSM, Fellowship of Trinity College London (FTCL) of Trinity College London, or had a master’s degree in music, or were professional pianists with extensive concert experience.

### 2.2. Experimental Setup and EMG Recording

The experiment included three scales (C major, A major, C major arpeggio) spanning over 4 octaves and two pieces (J. S. Bach’s Prelude in C major, from The Well-Tempered Clavier, Book I (BWV 846), R. Schumann’s “Happy Farmer” from Album for the Young (Op. 68 No. 10)), and they were collectively referred to as the five tasks.

The participants were required to perform the five tasks at two tempo (fast/slow) and force levels (soft/loud), that is, four combinations: fast and loud, fast and soft, slow and loud, and slow and soft. The four manners of playing were referred to as the four styles. In the experiment, we did not set up any hard standards but allowed the participants to interpret according to their natural playing manner. Tempo and force are two of the basic elements in musical expressions. All the musicians were well trained to flexibly adjust the tempo and force in performance to meet the requirements of the pieces and to realize their artistic expression. Such adjustment and adaptation requires precise control, and we were more likely to identify fine modulation on the muscle control modules. During the experiment, each scale with one style was repeated twice, while each piece with one style was only played once. One repetition was called one trial, and the order of the trials in the experiment was random to keep trials independent from each other.

**Figure 1:**
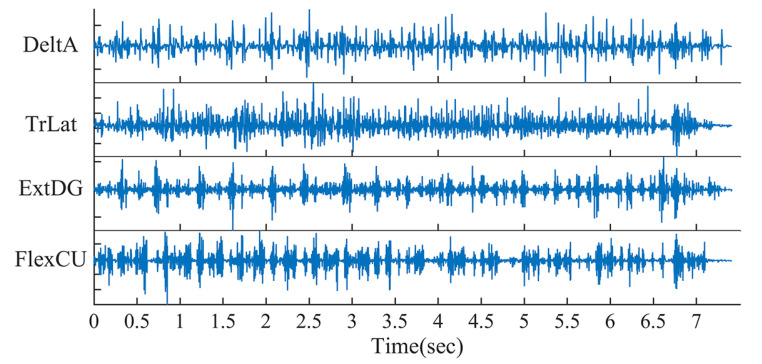
Sample EMG signals. This is an example of the EMG signals recorded from two upper-arm muscles (DeltA and TrLat) and two forearm synergies (ExtDG and FlexCU).

Participants played on a KORG KROSS 2 88-key keyboard (KORG, Tokyo, Japan). EMG signals were recorded from fourteen muscles on each side using wireless sensors (Trigno, Delsys, MA, USA; Noraxon, Scottsdale, AZ, USA). The two EMG recording systems were synchronized with Vicon system (Vantage, Vicon, Oxford, UK). The fourteen recorded muscles included six muscles on the shoulder and upper arm: trapezius major (TrapMaj), infraspinatus (Infrasp), anterior part of deltoids (DeltA), medial part of deltoids (DeltM), long head of biceps (BicLong) and lateral head of triceps (TrLat), and eight muscles on the forearm: brachioradialis (BrRad), pronator teres (PronTer), flexor carpi radialus (FlexCR), flexor carpi ulnaris (FlexCU), flexor digitorum (FlexDG), extensor carpi radialis longus (ExtCRL), extensor carpi ulnaris (ExtCU) and extensor digitorum (ExtDG). Figure 1 is an example of the EMG signals, including two upper-arm muscles and two forearm muscles.

### 2.3. EMG Preprocessing

At the beginning, we removed the trials containing dropped EMG channels and cut out the part with data loss longer than 0.5 s by manual inspection. The scales and pieces we included were simple for expert pianists, and the two tempo and force levels could be clearly distinguished even with part of the trial. Meanwhile, we recorded 32 trials for each subject, and the limited fluctuation brought by cut trials would not influence the general conclusion derived. The two trials from the same task and style of each subject were concatenated into one. From each concatenated trial, one set of muscle synergy was extracted. We first removed power line interference (PLI), background noise and their harmonics with the noise reduction algorithm proposed in [42]. We then band-pass filtered the signal between 20 Hz and 400 Hz to remove low-frequency motion artifacts and high-frequency noise, and then removed the baseline offset. Afterwards, we rectified the signal and low-pass filtered the signal at 40 Hz to get the envelope. With the low-pass filtering completed, we subtracted the minimum value of each channel to remove the static noise and integrated the signal with a 20-millisecond window for smoothing. Finally, we removed the spikes and normalized the signal by variance.

### 2.4. Muscle Synergy Extraction

We extracted the muscle synergies with an RLVM algorithm [38], which could reveal a set of latent variables with a smaller dimension from high-dimensional observed data. The use of RLVM for extracting inhibitory muscle synergy components has been previously proposed and explored by Guo and colleagues (X Guo, S. Huang, B. He, C. Lan, J. J. Xie, K. Y. S. Lau, T. Takei, A. D. P. Mak, R. T. H. Cheung, K. Seki, V. C. K. Cheung, and R. H. M. Chan, under review). Given recorded *N*-channel EMG $y_{t}$ at each time point *t*, it could be predicted with the following equation:

(1) $$y_{t}\approx f\left(Wc_{t}+b\right)$$

where f(.) is a parametric non-linearity, *W* is the N×M coupling matrix with each column as one muscle synergy, $c_{t}$ is the latent variable known as activation coefficients at time point *t*, and b is the bias. The RLVM algorithm only has a non-negative constraint on the activation coefficients $c_{t}$ and no such constraint on muscle synergy matrix *W*. In the model, an auto-encoder with a rectified linear unit (ReLU) function is first utilized to learn an optimized set of model parameters *W* and b. Afterwards, the optimized parameters learnt from the auto-encoder are used to initialize a maximum marginal likelihood (MML) algorithm to estimate the final *W* and b and infer the latent variables $c_{t}$ [38]. In order to make good use of former knowledge, we employed the synergies extracted using the NMF algorithm as the initialization of RLVM. Meanwhile, as the NMF algorithm has no bias term, we deleted the b from the model to avoid the possible false negative arising from the offset introduced by bias. And, the original EMG data could be reconstructed with *W* and $c_{t}$. The reconstruction level is quantified using the $R^{2}$ value. It implies the explained variance and is calculated using the following equation:

(2) $$R^{2}=1-\frac{SS_{res}}{SS_{tot}}$$

where $SS_{res}$ refers to the sum of squares of residuals, and $SS_{tot}$ refers to the total sum of squares. The $R^{2}$ value was utilized to decide the number of synergies to be extracted.

**Figure 2:**
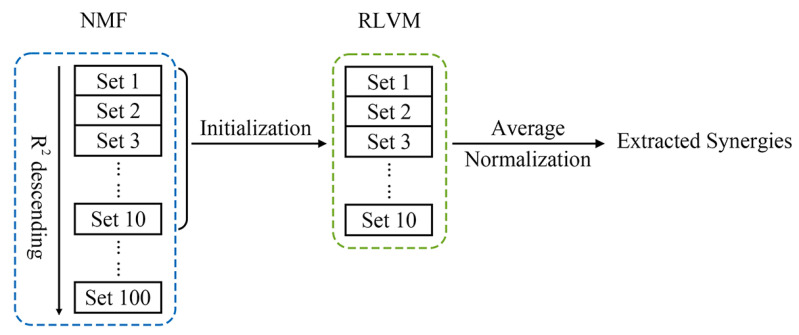
Muscle synergy extraction using RLVM algorithm with NMF synergies as initialization.

The NMF algorithm was first applied to the EMG data and repeated 100 times. The 10 repetitions with the highest $R^{2}$ values were used to initialize the RLVM model (blue dotted box in Figure 2). Using the 10 sets of NMF synergies, 10 corresponding sets of RLVM synergies were extracted (green dotted box in Figure 2). The dimensionality of the synergy set was decided as the minimum number of muscle synergies to ensure that the 10 sets of RLVM synergies all met an $R^{2}$ threshold of 0.8. According to our results, the ten RLVM synergy sets had very close $R^{2}$ values (difference in $R^{2}<10^{-4}$). Thus, the average synergy set across the 10 repetitions was employed for further analysis and was normalized by variance (Figure 2).

### 2.5. Muscle Synergy Clustering

**Figure 3:**
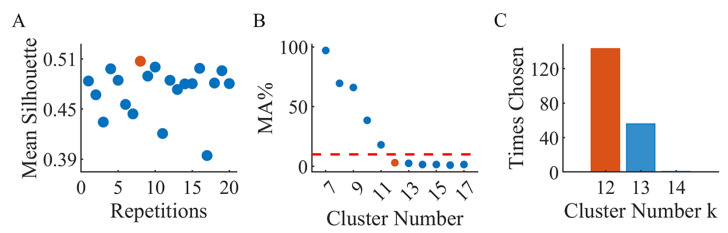
Clustering muscle synergies. (**A**) The best repetition with the highest mean silhouette value is marked with an orange dot. (**B**) The cluster number chosen according to the threshold of MA% is marked with an orange dot. The red dashed line marks out the threshold of MA%=10%. (**C**) The most chosen cluster number in 200 repetitions is marked out with an orange bar.

We first pooled the synergies from all the subjects, tasks and styles together but separated the left and right side. In the clustering strategy, the k-means clustering algorithm was repeated 20 times, and the repetition with the highest mean silhouette value was chosen (Figure 3A). Ideally, each synergy from one synergy set should be exclusively assigned to individual clusters. However, we also occasionally had two or three of the synergies end up being assigned the same one due to the high diversity. We refer to such condition as the mutual assignment (MA) of synergies. Considering this situation, the cluster number was decided to be the minimum number to ensure that the percentage of trials involving an MA of synergies would be less than 10% (MA%<10%), as shown in Figure 3B. The process was repeated 200 times, and the most selected cluster number was employed to obtain the final optimal clustering results (Figure 3C). At last, we re-classified the clustered synergies according to the styles for further analysis.

### 2.6. Proportion of Inhibitory Components

We first employed a general feature named as the proportion of inhibitory components calculated as the sum of absolute weights of the inhibitory components over the sum of absolute weights of all the components in the synergies. In this way, we were able to assess the contribution of inhibitory components in each synergy. In the analysis on age- and expertise-related changes in proportion, we applied the Kruskal–Wallis test to assess whether there was any significant difference between groups considering the unbalanced sample size. In the style-related analysis, we first paired the extracted features according to the style of interest. For instance, when studying differences across the tempo levels and features from the same force level, the same scale/piece and same subject but two different tempo levels would be paired. The features from two tempo levels must be dependent with each other, so we needed to employ a paired test. We also found that the extracted proportion was not normally distributed in all styles according to the Anderson–Darling test. Thus, we employed the Wilcoxon signed-rank test to assess whether there were any significant changes in proportion.

### 2.7. Modulation on Individual Inhibitory Components

In addition to the general feature proportion, we furthered our analysis by investigating which specific inhibitory components were modulated in style switching. Muscle channels having negative average value across all the synergies within the cluster in at least one style in comparison were regarded as “inhibitory components” and analyzed. We first paired the synergies in each individual cluster according to the style of interest. The weights of synergies from low-tempo (slow) trials were then subtracted from those of synergies from high-tempo (fast) trials to study tempo-related modulation, and the weights of synergies from the high-contact-force (loud) trials were subtracted from those of the synergies from the low-contact-force (soft) trials to study force-related modulation. The distribution of weights of most components was leaned and not normal with the Anderson–Darling test; we applied Wilcoxon signed-rank test to the weights of the inhibitory components to identify significant changes. Clusters having fewer than 10 pairs of data were regarded as uncommon and excluded from analysis.

## 3. Results

### 3.1. Clustered Synergies

**Figure 4:**
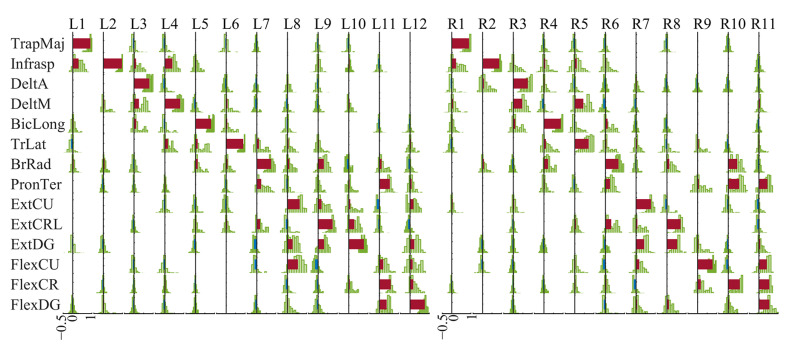
Centroids of synergy clusters. Brown bars represent positive weights, blue bars represent negative weights, and green histograms show the distribution of the weights of each channel in each cluster. L1 to L12 are from the left side, while R1 to R11 are from the right side. Only components with significant non-zero median weights across instances within a cluster are shown (p<0.05, Wilcoxon signed-rank test).

**Figure 5:**
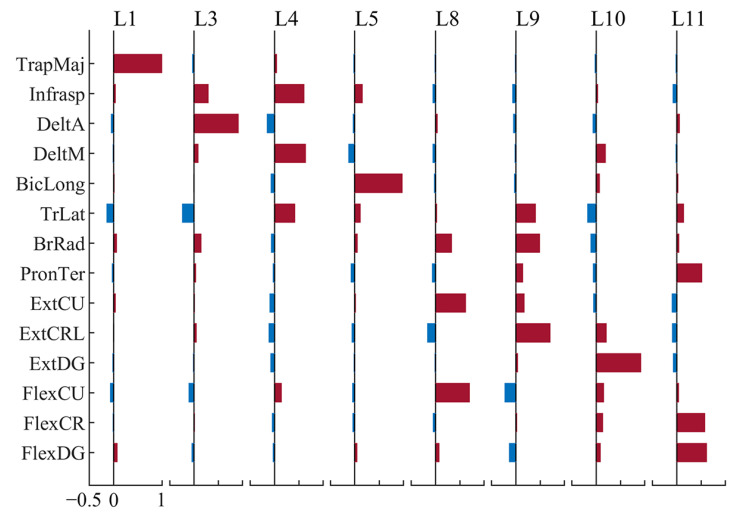
Example of synergies extracted from one trial of Exp04. Brown bars represent positive weights and blue bars represent negative weights.

We found 12 clusters on the left side and 11 on the right side, as shown in Figure 4. Among them, L1 to L6 and R1 to R5 are upper-arm synergies, and L7 to L12 and R6 to R11 are forearm synergies according to their most dominant components. From the distribution of weights of muscles (green histograms in Figure 4), we could tell that the muscle synergies extracted with the RLVM did have inhibitory components, which had commonly negative weights across instances in the clusters, such as FlexCU in L9. Though the weights of some components appeared to be randomly positive or negative across subjects and trials and failed to reject the null hypothesis of zero-median, displayed as blanks in Figure 4, components with a negative value are relatively common in individual synergy sets (Figure 5). On average, each synergy had around six channels (6.5758 on the left side, 6.5562 on the right side) being negative. However, most of them were minor components, with the 75th percentile of absolute weights being 0.098 on the left side and 0.10 on the right side. Meanwhile, Figure 5 also shows that subjects selected part of the identified typical synergies to complete the task and meet the requirement, instead of using all of them.

### 3.2. Changes in Proportion Related to Ages, Expertise and Styles

**Figure 6:**
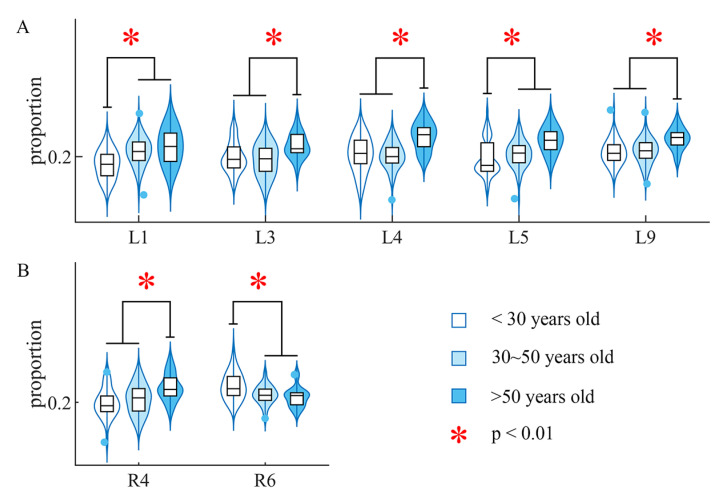
Difference in proportion of inhibitory components across ages. The black lines and red asterisks mark out the group having significant difference from the other two groups. (**A**) Results of proportion comparison on the left side. (**B**) Results of proportion comparison on the right side.

In the age-related comparison of the proportion, the subject over fifty years old had a significantly higher proportion of inhibitory components (p<0.01, Kruskal–Wallis test) than the other two younger groups in L3, L4 and L9 on the left side, and R4 on the right side. Meanwhile, the group under thirty had a significantly lower proportion in L1 and L5 on the left side (Figure 6).

**Figure 7:**
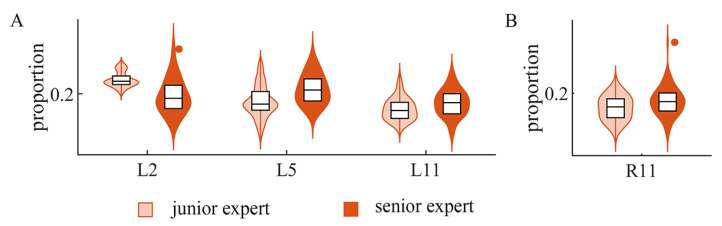
Difference in proportion of inhibitory components on expertise. (**A**) Results of proportion comparison on the left side. (**B**) Results of proportion comparison on the right side.

In the expertise-related comparison, our results in Figure 7 show that the senior expert group had a higher proportion of inhibitory components in L5 and L11 on the left side and R11 on the right side, but a lower proportion in L2 on the left side and R9 on the right side (p<0.01, Kruskal–Wallis test). In general, senior experts showed more changes on the left side and mostly had a higher proportion of inhibitory components.

**Figure 8:**
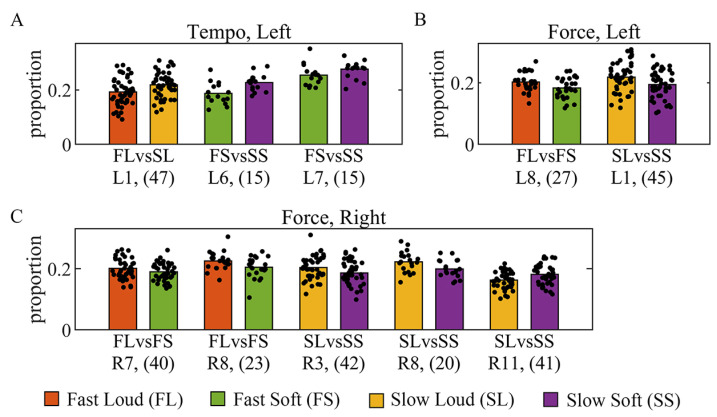
Difference in proportion of inhibitory components across styles. The height of the bars show the mean proportion, and each black dot represents the proportion of one synergy. The numbers in brackets are the sample size, and the two compared styles share the same sample size. (**A**) Tempo-related changes on the left side, (**B**) force-related changes on the left side, (**C**) force-related changes on the right side. Difference was found to be significant with p<0.05 using Wilcoxon signed-rank test. No significant difference was found when tempo changed on the right side.

In style-related comparison, low contact force (soft) and high tempo (fast) generally led to a lower proportion of inhibitory components. On the left side, subjects had a lower proportion at fast loud in L1, fast soft in L6 and L7 when tempo changed (Figure 8A), and fast soft in L8 and slow soft in L1 when force changed (Figure 8B). On the right side, subjects had lower proportion at fast soft in R7 and R8, at slow soft in R3 and R8 (Figure 8C).

### 3.3. Modulation of Inhibitory Components across Styles

**Figure 9:**
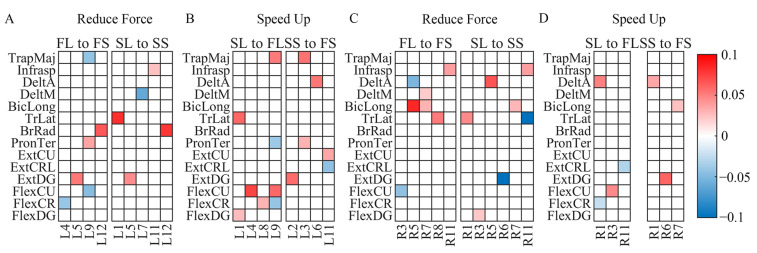
Modulated inhibitory components across styles. The heat-maps show the average difference in the inhibitory components having significant changes between styles (p<0.05, Wilcoxon signed-rank test). A blue box indicates that the component became more negative, and a red box indicates that it became less negative. FL means Fast and Loud; FS means Fast and Soft; SL means Slow and Loud; SS means Slow and Soft. (**A**) Modulation on the left side when subjects reduced force. (**B**) Modulation on the left side when subjects sped up. (**C**) Modulation on the right side when subjects reduced force. (**D**) Modulation on the right side when subjects sped up.

Generally, the more inhibitory components became less negative both when reducing force and speeding up. When subjects reduced the force, seven inhibitory components on the left side and eleven on the right side became less negative, while only four on the left side and three on the right side became more negative. When subjects sped up, ten inhibitory components on the left side and five on the right side became less negative, while only four on the left side and two on the right side became more negative (Figure 9).

**Figure 10:**
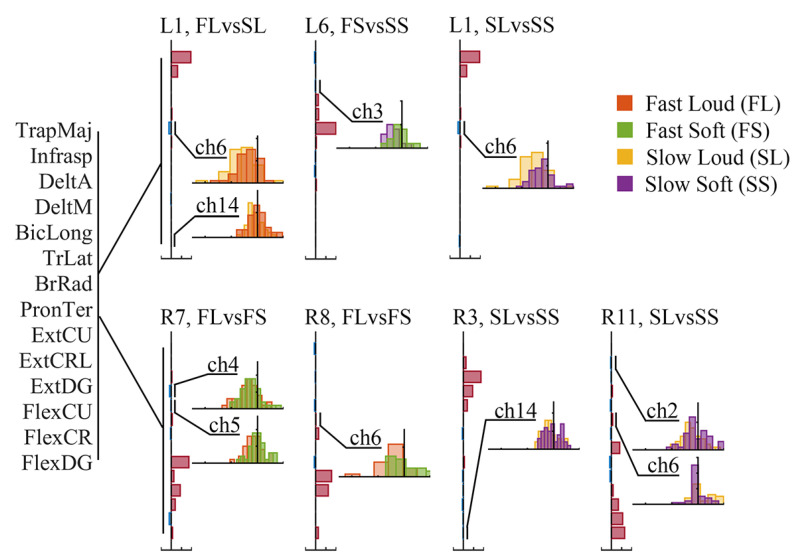
Modulated inhibitory components in synergies having proportion changes. The muscle names of each EMG channel are shown on the left side of the figure. The horizontal bar charts show the normalized centroids of corresponding clusters, with brown bars representing positive values and blue bars representing negative values. The vertical histograms show the distribution of weights, using one color representing one style.

Furthermore, we had a close look at those synergies that had different proportions between tempo/force levels, that is, L1, L6, L7 and L8 on the left side, along with R3, R7, R8 and R11 on the right side (Figure 8). We found two synergies on the left and four on the right also had inhibitory components commonly modulated (Figure 10). On the left side, TrLat (channel 6) and FlexDG (channel 14) in L1, and DeltA (channel 3) in L6 was more negative at low tempo (slow); TrLat (channel 6) in L1 was also more negative high force level (loud). On the right side, DeltM (channel 4) and BicLong (channel 5) in R7, TrLat (channel 6) in R8, FlexDG (channel 14) in R3 and Infrasp (channel 2) in R11 were more negative at high force levels (loud).

## 4. Discussion

This study identified inhibitory components of motor modules from EMG recordings that were neglected by the commonly utilized NMF algorithm, and revealed the modulation of these components related to ages, expertise and variant force and tempo requirements in a complex motor task.

With the same dimensionality, the $R^{2}$ values achieved by the RLVM algorithm and the NMF were approximately the same, while the RLVM was able to detect the inhibitory components with negative weights. Our results reveal that the inhibitory components were relatively common in the muscle synergies, while most of them had minor weights compared with the positive components (Figure 4). This finding is consistent with [25], which mentioned that few neurons had suppressive effects on the muscles. Meanwhile, we also found that the most obvious inhibitory component in the synergies of flexors would usually be extensors, and vice versa, such as L9, L11, R7 and R11. This is consistent with the intuition and knowledge that the contraction of one muscle could be associated with the suppression of its antagonist [43].

We found that in most cases, the group over 50 years of age had a higher proportion of inhibitory components in the relevant synergies, but the group under 30 had lower (Figure 6). The increase in proportion of inhibitory components could also be interpreted as a decrease in the proportion of positive components in the older group, which could probably be induced by a reduction in the ratio of excitatory to inhibitory synapses proposed in [32]. Previous muscle synergy analysis on aging [44] proposed a higher level of activity of recruited muscles within each synergy in older groups in walking balance. Guo et al. [45] further reported age-related modulation in specific positive muscle components during overground walking. Our conclusion on age-related changes in proportion also revealed that the structure of synergy could be fine-tuned by aging, which is consistent with former works showing that muscle synergies can be fine-tuned over developmental stages [45,46]. Furthermore, our work focused on inhibitory components, long neglectedin previous research, and provided additional possibilities to muscle synergy analysis.

Meanwhile, we also found that the inhibitory components were fine-tuned according to the different expertise levels. On the left side, senior experts generally had a higher proportion of inhibitory components in two synergies (Figure 7A) and had a lower proportion in one synergy on the right side (Figure 7B). Multiple research studies have reported modulation in the individual muscle components of synergies after a training session (four weeks in [47]) or even just short-time practice (twenty forty-five-second-long practice trials in [34]). Our results on changes in the proportion implying modulation in inhibitory components are consistent with the findings on the positive components. Furthermore, we found that the influence of expertise was more obvious on the left side. It may arise from the fact that all of the ten subjects were right dominant, as well as the training ways of this specific experiment task—piano playing. In many piano pieces, the right hand is responsible for rendering the melody, which usually requires a more complex note sequence compared with the left hand. In other words, the two hands of pianists are not trained equally, which may also account for the distinction between the two sides.

Another important finding of our study is that the inhibitory components were also modulated while adapting to variant requirements. High-tempo (fast) and low-contact force (soft) led to a lower proportion of inhibitory components. Furthermore, the weights of most inhibitory components found to be commonly modulated also became less negative when reducing force or speeding up (Figure 9 and Figure 10). In research on *Drosophila* larvae, it was discovered that the activation of a group of inhibitory premotor neurons would lead to the relaxation of body-wall muscles and slow the larvae down [48]. It may be the reason why the inhibitory components became less negative at a higher tempo, where the muscles became less relaxed and tended to contract to speed up. A study based on treadmill walking revealed that the contribution of specific muscles increased/decreased as speed changed [49]. It also proposed that the changes in muscle contributions might relate to the proportions of fast and slow muscle fibers [50,51], which may also work for modulation in the muscle components appearing inhibitory. With regard to force changes, ref. [52] found an increase in the contribution of two positive muscle components within one synergy as force increased. Thus, the contribution should decrease at a high force level in inhibitory components. However, in contrast, we found a decrease at a low force level (soft). The observation that soft playing in piano performance is more challenging than playing loudly may be attributed to the greater control required for softer dynamics.

There are several limitations in our research, including small and unbalanced sample sizes in the age- and expertise-related analyses, which restricted our ability to use more than just general features like the proportion of inhibitory components. Despite these constraints, we successfully identified the inhibitory components and demonstrated their modulation in response to dynamic force–tempo variations. While this initial study on inhibition in the synergistic control of muscles relied solely on surface EMG recordings and therefore could not fully elucidate the underlying neural mechanisms, it establishes a foundation for more comprehensive future investigations that could significantly deepen our understanding of motor control.

## 5. Conclusions

Our study turns attention to the inhibitory components in motor modules, which have long been neglected in muscle synergy analysis. Our results reveal that minor weights, aging and expertise shaped these inhibitory components. They were also modulated during adaptation to variant requirements, which further demonstrates that the inhibitory components also contribute to the fine control of muscles in a complex motor task. Our results imply that introducing inhibitory components may be helpful in controlling a system with a high number of degrees of freedom. This finding would benefit robotic control and may improve the functionality of motor prostheses.

## Data Availability

All the data included in this article are available from the corresponding author upon request.

## Abbreviations

The following abbreviations are used in this manuscript:

EMG	Electromyography
RLVM	Rectified Latent Variable Model
CNS	Central Nervous System
NMF	Non-negative Matrix Factorization
TrapMaj	Trapezius Major
Infrasp	Infraspinatus
DeltA	Anterior part of Deltoids
DeltM	Medial part of Deltoids
BicLong	Long Head of Biceps
TrLat	Lateral Head of Triceps
BrRad	Brachioradialis
PronTer	Pronator Teres
FlexCR	Flexor Carpi Radialus
FlexCU	Flexor Carpi Ulnaris
FlexDG	Flexor Digitorum
ExtCRL	Extensor Carpi Radialis Longus
ExtCU	Extensor Carpi Ulnaris
ExtDG	Extensor Digitorum
PLI	Power Line Interference
ReLU	Rectified Linear Unit
MML	Maximum Marginal Likelihood
MA	Mutual Assignment
